# Institutional Housekeeping

**Published:** 1912-01-06

**Authors:** 


### INSTITUTIONAL HOUSEKEEPING.

(Contributions invited: Workers' Questions will be welcome.)

### How to Avoid Wasting Coal.

Fuel must of necessity be one of the most expensive items in an institution, and never ceasing supervision is needed to control the amount used so as to obtain the largest amount of heat at the smallest possible cost.

In hospitals it is not usually the housekeeper's duty to order the coal, but that of the secretary or house governor; but she should observe the kind of coal sent, and the manner of delivery no less carefully than its use in the wards, so that if she receives complaints that the quantity required is increasing, or that the coal is not satisfactory, she may be able to find out the cause.

It is usual to order the coal straight from the colliery in large quantities, the amount got in at one time depending on the heating apparatus in the institution.

Where furnaces are to be kept going with large boilers to heat, it is necessary to stock two kinds of coal. The hard steam coal suitable for boilers, cooking, etc., gives out a powerful heat, does not burn rapidly, is inexpensive, and makes little smoke or dust, but it requires a good draught to ignite it, so that it is not suitable for an open fire. For this purpose coal with a dusty, dull appearance should be avoided; it will make much ash and give out little heat, nor is cheap coal economical for open grates. It often contains slatey substances and makes wasteful ash.

The softer, brighter varieties known as Wallsend, Silkstone, and Derby brights are best for grates that have little draught, but these are more expensive and burn more quickly than the hard steam coals, and should not be used for furnaces. If the softer coal must be used for stoking purposes, it is economical to mix selected coke with it. Coal and coke once ignited give the maximum amount of heat and last longer alight than any other kind of fire.

The coke should be delivered in pieces about the size of a large egg and should be mixed with an equal proportion of coal. Coke is very light, and is therefore comparatively much cheaper than coal. Its use will prevent the chimneys from fouling so quickly. Coal being cheaper in summer, it is the practice in all economical institutions to get in their large stores at this season. A great deal depends on careful observation at the time of delivery, and the tedious duty of checking the weight and number of carts should be assigned to a thoroughly responsible official. The pieces ought not to be too large, or breaking them up causes much dust, nor should the delivery of large quantities of slack with the coal be permitted. It should be stored in covered sheds fitted with locks; and if the quantity stored be very large, these sheds should not be too near the building, as a certain amount of gas escapes from it.

The coal stores should be well lighted, so that the coal is exposed to view when being accepted and to avoid wasteful accumulations of dust. In a good-sized institution there should always be 100 tons or more in store, but this should be used in regular sequence, for if coal is kept for a long time it deteriorates and crumbles when used.

It is unavoidable that a proportion of small coal and dust should accumulate, and this should be kept in a separate heap or shed. A portion of this refuse must be regularly distributed, and before each delivery of coal it should be entirely used up. The large pieces should be used for the ward fires, the next size for the kitchen and scullery, and the small coal and dust, well damped, can be utilised for boilers, furnaces, etc.

Coals are delivered to the wards by the porters, and it is the housekeeper's duty to supervise them so that suitable pieces are provided for every department.

By observation it can be ascertained what amount of coal will keep a fire up for a given time in order to keep the wards at a given temperature, and coal should be distributed thus daily, due allowance being made for fluctuations in weather. In stoking a fire all the small coal and dust must be used, and banking it well up with damped small coal will make it last for hours without attention. If not much heat is required, as, for example, between meal times in the dining-room or servants' hall, briquettes may be used, as they burn slowly and are less expensive than coal, but do not give out much heat.

Slow-combustion grates are most economical, and a little money spent on the grates would often save many pounds in the consumption of coal. In all open grates solid brickwork round the grate and the use of firebricks in the fireplaces are indispensable factors in the economy of fuel. The front of the grate should be two-thirds larger than the back to throw out heat. The bars should be vertical to make combustion slower and to prevent coal from falling through. The throat of the chimney should be set well forward to prevent loss of heat.

In the kitchen all flues should be swept at least once a week, twice is better, and *every day* the soot must be removed from the top of the oven under the hot plate, as soot is a very bad conductor of heat. If the housekeeper hears that the ovens will not get hot, it is an easy matter to remove a ring from the hot plate and scrape with a poker, and she will most likely find an accumulation of soot.

Kitchen refuse may be dried and burnt and will keep the fire up for a long time; it should be placed in the back of the fire, the stove shut up, and the dampers drawn out to create a draught. It is much more wholesome to burn refuse than to throw it in the dustbin, and this can be done every afternoon should the fire not be required to sit by.

The saving of fuel lies in so many hands it is most difficult to prevent waste, and it needs co-operation on the part of the secretary or house governor with the matron and housekeeper to detect any leakage and to make use of the fuel to the best advantage.

